# Insights into Drought Stress Signaling in Plants and the Molecular Genetic Basis of Cotton Drought Tolerance

**DOI:** 10.3390/cells9010105

**Published:** 2019-12-31

**Authors:** Tahir Mahmood, Shiguftah Khalid, Muhammad Abdullah, Zubair Ahmed, Muhammad Kausar Nawaz Shah, Abdul Ghafoor, Xiongming Du

**Affiliations:** 1 State Key Laboratory of Cotton Biology, Institute of Cotton Research (ICR), Chinese Academy of Agricultural Sciences (CAAS), Anyang 455000, China; tahirmtaha@hotmail.com; 2 Department of Plant Breeding and Genetics, Pir Mehar Ali Shah Arid Agriculture University, Rawalpindi 46000, Pakistan; shiguftah@outlook.com (S.K.); Ayda3249@yahoo.com (M.A.);; 3 National Agriculture Research Center (NARC), Pakistan Agriculture Research Council, Islamabad 44000, Pakistan; 4 Member of Plant Sciences Division, Pakistan Agricultural Council (PARC), Islamabad 44000, Pakistan; 5 School of Agricultural Sciences, Zhengzhou University, Zhengzhou 450001, China

**Keywords:** cellular stress signaling, drought stress responses, functional genomics, gene identification tools, drought tolerance, cotton molecular genetic basis, *Gossypium*

## Abstract

Drought stress restricts plant growth and development by altering metabolic activity and biological functions. However, plants have evolved several cellular and molecular mechanisms to overcome drought stress. Drought tolerance is a multiplex trait involving the activation of signaling mechanisms and differentially expressed molecular responses. Broadly, drought tolerance comprises two steps: stress sensing/signaling and activation of various parallel stress responses (including physiological, molecular, and biochemical mechanisms) in plants. At the cellular level, drought induces oxidative stress by overproduction of reactive oxygen species (ROS), ultimately causing the cell membrane to rupture and stimulating various stress signaling pathways (ROS, mitogen-activated-protein-kinase, Ca^2+^, and hormone-mediated signaling). Drought-induced transcription factors activation and abscisic acid concentration co-ordinate the stress signaling and responses in cotton. The key responses against drought stress, are root development, stomatal closure, photosynthesis, hormone production, and ROS scavenging. The genetic basis, quantitative trait loci and genes of cotton drought tolerance are presented as examples of genetic resources in plants. Sustainable genetic improvements could be achieved through functional genomic approaches and genome modification techniques such as the CRISPR/Cas9 system aid the characterization of genes, sorted out from stress-related candidate single nucleotide polymorphisms, quantitative trait loci, and genes. Exploration of the genetic basis for superior candidate genes linked to stress physiology can be facilitated by integrated functional genomic approaches. We propose a third-generation sequencing approach coupled with genome-wide studies and functional genomic tools, including a comparative sequenced data (transcriptomics, proteomics, and epigenomic) analysis, which offer a platform to identify and characterize novel genes. This will provide information for better understanding the complex stress cellular biology of plants.

## 1. Introduction

Global warming and climate change adversely affect agricultural production. Erosion of genetic diversity for drought tolerance in major crops is a threat to food security. Abiotic stresses are major threats, and collectively led to 73% decline in cotton production worldwide [1]. Drought refers to low water availability for the long-period of time, and affects crop production [2]. Drought tolerance is a complex trait involving multiple genes associated with cellular signaling pathways which modify several physio-morphological, and molecular responses. Plant cell membranes perceive stress signals and stimulate various self-activated and hormone-dependent signaling mechanisms [3]. Mitogen-activated-protein-kinase (MAPK) networks are involved in stress signaling and activate several stress-responsive proteins [4]. In stress signaling pathways, calcium (Ca^2+^) is a common second messenger, controls many physiological processes in plants. The cytoplasmic Ca^2+^ concentration varies in response to drought stress and various hormones such as abscisic acid (ABA), jasmonic acid (JA), and ethylene [5]. Under high concentrations, ABA interacts with SnRK2 proteins, which subsequently initiate molecular and physiological responses to drought stress [6,7,8]. Jasmonic acid (JA) and its derivatives also activate signaling pathways similar to ABA [9]. Overproduction of reactive oxygen species (ROS) also triggers defense mechanisms and excessive amounts of ROS scavenged by enzymatic and non-enzymatic defense machinery in plants [10].

Following the successful transduction of stress signals, plants actively adopt drought recovery mechanisms. Tolerant plants are able to resume growth and overcome the growth deficit induced by drought. Cotton has developed numerous morpho-physiological approaches, such as photosynthetic response [11], osmotic adjustment, stomatal regulation, low leaf water loss, high relative water contents (RWC), and enlarged tap roots [12]. These features contribute to drought tolerance through a multigenic effect. Genetic statistics and improvements of physio-morphological characters are important to reduce the effects of drought. Alterations in physio-morphological and biochemical traits have vital roles in maintaining favorable water balance in plant cells and tissues.

Genome modification technologies and transgenic approaches have been employed to develop drought-tolerant crops overexpressing transgenes that are important for plant physiology. Targeted genome editing with the CRISPR/Cas9 system has been utilized to modify the genome to obtain more stable and heritable mutations [13]. Genome-wide studies have been performed to explore stress-related candidate regions and genes for drought tolerance. Various drought-related quantitative trait loci (QTL) clusters and hotspots have been mapped in cotton. Several QTLs for abiotic stress, especially drought, have been identified using single nucleotide polymorphisms (SNPs) in genome-wide association studies (GWAS). Meta-analyses can be performed to identify common QTLs for drought-related traits [2]. Whole genome sequencing and re-sequencing of allotetraploid and diploid cotton species provide information in the biologically active states of DNA [14]. Fine- and high-density genetic maps, transcript abundance, epigenetic modifications, and SNP array platforms can also be used, as reported for other model plants (rice and *Arabidopsis*). These approaches serve as a platform for gene mapping, isolation, and cloning for drought tolerance. Moreover, the identification of novel genes can be facilitated by high-throughput marker development for stress tolerance in plants.

This review focuses on the cellular and molecular signaling networks and drought coping adaptations in plants to overcome the impact of drought stress. The use of functional genomics to overcome drought stress is also discussed. Furthermore, this review provides an overview of the genetic basis of drought tolerance in cotton, with a focus on QTLs and candidate abiotic stress tolerance genes in cotton, which might be employed for novel cotton breeding in the future.

## 2. Cellular and Molecular Signaling Pathways of Drought Stress Tolerances

**Figure 1:**
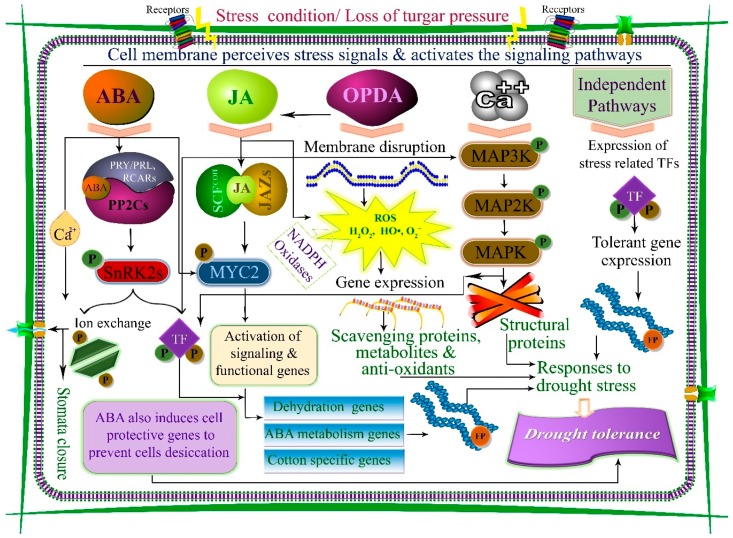
Drought-induced cellular and molecular signaling pathways to enhance drought tolerance in plants. The cell membrane perceives stress signals and triggers signaling. In the presence of abscisic acid (ABA), a complex of PRY/PRL, RCARs, and PP2Cs is formed, which dissociates PP2Cs from SnRK2 and activates NnRK2 protein (P). SnRK2 is auto-activated when separated from PP2C. Activated SnRK2 triggers and regulates molecular and physiological responses. Similarly, jasmonic acid (JA) is engaged with the jasmonate-zim domain (JAZ) in a complex with SCF and TFs (MYC2), and activates stress- responsive genes. Overproduction of reactive oxygen species (ROS) in response to oxo-phytodienoic acid (OPDA) and JA activates scavenging genes and act like a stress-signaling unit. Calcium (Ca^2+^) interacts with mitogen-activated protein kinase (MAPK) cascade proteins to activate transcriptional factors and signaling genes. Finally, functional proteins (FP) are synthesized for drought-stress responses.

Plants have evolved several cellular and molecular signaling pathways to activate and regulate defense mechanisms against biotic and abiotic stresses. Drought tolerance is a complex trait involving multi genes in physio-morphological, molecular, and biochemical processes and pathways (Figure 1). However, these mechanisms have not been fully explored in cotton, and may provide the potential to improve its drought tolerance.

### 2.1. Mitogen Activated Protein Kinase (MAPK) Signaling Pathway 

Plants have acquired various adaptations against environmental stresses through numerous molecular networks, comprising stress sensing, signaling, and expression of stress-sensitive genes. The MAPK cascade is a vital tool established by plants to respond to abiotic and biotic stresses; it regulates responses by transducing signals in response to extracellular stimuli. Several processes, such as hormonal responses, developmental programs, cell division, proliferation, apoptosis, and other stress responses, are regulated by MAPK pathways, which are highly conserved and centrally regulated. Three diverse protein kinases: MAPK, MAPKK, and MAPKKK collectively comprise the cascade. The activation of these kinases involves sequential phosphorylation [4]. Activation of the MAPKKK protein results in phosphorylation of threonine or serine residues at the S/T-X3-5-S/T conserved motif located in the MAPKK activation loop [15]. Activation of MAPKK results in phosphorylation of MAPK on tyrosine and threonine in the activation loop of the T-X-Y invariant motif [16]. MAPK phosphorylates selected targets and controls the activity of phospholipases, microtubule-related proteins, cytoskeletal proteins, kinases, and other transcription factors (TFs), whose actions facilitate numerous responses (Figure 1) [8].

Recently, the ABA-activated MAP3K18 kinase was found to be involved in stomatal signaling and development in Arabidopsis [17]. *map3k18* mutant plants showed reduced stomatal index and larger stomatal size under normal growth conditions compared with the wild type. In addition, the *map3k18* mutant exhibited ABA-induced stomatal aperture and closure. Therefore, MAPKKK18 was hypothesized to participate in drought resistance by playing a virtual role in stomatal signaling under drought stress [18].

**Figure 2:**
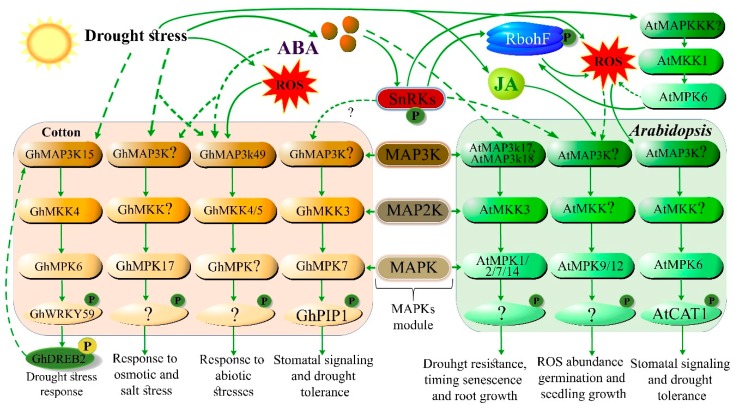
Drought-induced, ABA-dependent, ABA-independent MAPK signaling, and interaction between ABA, ROS, and MAPK signaling under drought stress in plants. ABA-regulates various MAPKs in cotton and Arabidopsis. ABA promotes drought sensing and signaling in plants. The different cascades are represented by different color schemes in the figure. Solid arrow lines denote established signaling mechanisms, while dashed arrow lines denote unestablished signaling pathways. ABA-activated SnRK2s (See Figure 1 for SnRK2 activation) trigger and phosphorylate downstream targets, such as respiratory burst oxidase homolog (RBOH) and various MAPKs. Activation of RBOH induces ROS production. ROS signaling and ABA signaling may overlap with MAPK factors, to interact and regulate drought tolerance. MAP3K17/18-MKK3-MPK1/2/7/14 is an ABA-induced complete MAPK cascade involved in stomatal signaling, senescence, and drought tolerance mechanisms in *Arabidopsis*. In addition, MKK1 activates MPK6 to positively regulate CATALASE1 (CAT1) for ROS abundance. In cotton, the drought- and ABA-induced MAPK cascade MKK3-MPK7-PIP1 is associated with stomatal signaling and drought tolerance. Another ABA-mediated MAPK module, MAPKKK49-MKK4/MKK5, is associated with abiotic stress responses. *GhMPK17* gene is a novel, well-characterized MAPK, which is associated with responses to osmotic and salt stresses in cotton. An ABA-independent and drought-mediated MAPK module (MAP3K15-MKK4-MPK6-WRKY59) regulates drought tolerance in cotton. Drought stress triggers the MAPKKK15 cascade, which phosphorylates the WRKY59 transcriptional factor. Interestingly, WRKY59 binds to the promoter of *DREB2* and regulates the expression of drought-sensitive genes. Meanwhile, it positively regulates the expression of *MAP3K15* by establishing a feedback loop, which regulates drought tolerance in cotton.

At a transcriptional level, the M3K18 promoter demonstrated higher promoter efficiency following stimulation with ABA in plant guard cells. These results indicated that, MAP3K18 interacts directly with key components of the ABA signaling module, such as SnRK2.6 kinase [19] and PP2C phosphatase ABI1 [17]. ABA engages PYR/PYL (Figure 1) and prevents the degradation of MAP3K18, which ensures the stability and activation of downstream kinase processes to trigger stress signaling modules [17]. Two research groups independently reconstructed the intact MAPK cascade regulated by ABA and initiated by MAP3K18 [20,21]. The complete cascade regulated by ABA, MAPKKK17/18-MKK3-MPK1/MPK2/MPK7/MPK14, has been reported to be involved in senescence [21] and stress-signaling mechanisms (Figure 2) [20]. Recently, the role of this cascade in drought resistance has also been reported [18]. A proximal homolog of *MAP3K17*, *MAP3K18*, was identified in *Arabidopsis*. The kinase activity of *MAP3K18* and *MAP3K17* is enhanced after ABA treatment [17,20]. Furthermore, a positive correlation exists between the *MAP3K18/MAP3K17* gene and the transcriptional level of the ABA signal transduction gene [20]. ABA also interacts with AtrbohF [22] and MAPKs which regulates the stomatal signaling under stress. Meanwhile AtrbohF also interacts with Ca^2+^ signaling and improves oxidative stress tolerance in *Arabidopsis* [23].

Various environmental stresses that activate MAPK signaling pathways in cotton have been reported. Recently, a new cotton *MAPM3K* gene, *GhMAP3K49*, was identified, which is significantly induced by ABA and ROS [24]. GhMAP3K49 interacts with GhMKK9 and GhMKK4 in a complete cascade. In this regard, it may be assumed that the GhMAP3K49-GhMKK9 or GhMAP3K49-MKK4 cascade is involved in ROS (H_2_O_2_) and ABA-mediated responses to various abiotic stresses. Overexpression of the *GhMKK3* gene in *Nicotiana benthamiana* induces drought resistance by controlling the rate of water loss, stomatal count, and stomal aperture, induced by ABA [25]. Notably, GhPIP1 and GhMKK3 interact with GhMPK7 to construct a drought-activated and ABA-functional MAPK module in cotton [20]. 

Another recent study showed that GhWRKY59 (transcription factor, WRKY) plays an important role in ABA-independent induced MAPK cascade phosphorylation. An established MAPK cascade comprising GhMAPKKK15-GhMKK4-GhMPK6 was found in cotton. GhWRKY59 actively controls MAPK activation and *GhMAPKKK* expression through feedback. GhWRKY59 binds to the *GhDREB2* promoter and regulates the expression of drought-sensitive genes. Furthermore, it positively regulates *GhMAP3K15* expression by establishing a feedback loop (Figure 2). Ectopic overexpression of *GhWRKY59* in *Arabidopsis* increased drought resistance compared to wild type. A novel GhMAP3K15-GhMKK4-GhMPK6-GhWRKY59 phosphorylation loop that regulates the GhDREB2-mediated and ABA-independent drought response in cotton has been identified [26]. 

Several MAPK-related genes have been identified in the *Gossypium raimondii* and *Gossypium hirsutum* genomes, which are intricately related to MAPK pathways and involved in response to various environmental stresses, including cold, heat, and drought (Table 1) [27]. Further functional investigations of MAPK signaling cascades will provide insight into their biological functions and roles in response to hormonal stress responses and interactions between members of the MAPK gene family.

### 2.2. Ca^2+^ Signaling Pathway

In higher plants, calcium (Ca^2+^) is an important regulator of several physiological and cellular biochemical processes. Ca^2+^ is a common second messenger in signal transduction pathways and controls many physiological processes in cotton. Cytoplasmic Ca^2+^ concentration changes in response to hormones, ABA, and drought stresses [5]. Three main classes of Ca^2+^ sensor molecules are involved in the detection and transmission of signals: (i) calmodulin (CaM) and CaM-associated proteins, (ii) calcineurin B-like proteins (CBLs), and (iii) calcium-dependent protein kinase (CDPK), which detect and transmit cellular signals. Calmodulin is acidic in nature. Ca-binding-protein comprises four EF motifs, structural domains (helix-loop-helix) that coordinate with calcium ions. Ca^2+^ binds to the EF hand motif, as changes in CaM promote catalytic activity of its own or target proteins. Various sensor genes for CaM and other Ca^2+^ -related transcripts exist for fiber elongation in cotton [28]. However, no such reports for drought tolerance in cotton exist, and only a few CDPKs have been characterized in cotton. In a previous study, the *GhCPK1* gene was identified for the first time to have a role in Ca^2+^ signaling related to fiber elongation [29]. Following sequencing of the *Gossypium raimondii* genome, 41 CDPK genes were identified [5]; CDPK genes are responsive to drought stress. Another group of specific Ca^2+^sensors are the CBL proteins, which decode Ca^2+^ transients and regulate specific CBL-interacting-protein-kinase (CIPK) family members in higher plants. Drought, salt, and ABA treatments induce *GhCIPK6*, and its over-expression was found to enhance drought stress tolerance [30]. Thus, subsequent response and downstream targets of proteins are induced by changes in Ca^2+^ concentrations, which transduce Ca^2+^ signals through CaMs, CDPKs, and CBLs during abiotic stresses, particularly drought stress (Figure 1).

### 2.3. Abscisic Acid (Aba)-Mediated Signaling Pathway

Plant hormones regulate stress-mediated signaling pathways; however, ABA signaling has been extensively studied in plants compared to other hormones. ABA is a key signaling regulator in many vital plant processes (defense, physiology, growth, and development). It also plays an important role in germination and seed dormancy in response to various abiotic and biotic stresses [31,32]. Approximately 10% of signaling genes are regulated by ABA in *Arabidopsis thaliana* [33]. In ABA-dependent pathways, ABA is responsible for the expression of stress responsive genes. ABA receptor elements have been identified in several parts of the cell, including the cytosol, nucleus, plasma membrane, and chloroplast envelope, which are involved in ABA signal transduction. At low levels of ABA, protein phosphatase 2C (PP2C) inhibits the effects of sucrose none-fermenting 1-related protein kinase 2 (SnRK2), which promotes dephosphorylation. Shortly after plants are exposed to drought stress, ABA level increases in the cell, binds to PYL/PYR/RCARs, and inactivates PP2Cs. SnRK2 proteins are activated automatically in the absence of PP2C. Activation of SnRK2s initiate ABA-induced molecular and physiological responses to drought stress (Figure 1) [6,7,8].

Recent studies have illustrated that expression of ABA-mediated genes has increased significantly in various plants species, especially in *Arabidopsis* and cotton. ABA regulates the transcription of MAPK genes in plants. The ABA signaling pathway regulates gene expression, transcriptional modifications, transcriptional processes, and stability [34]. Modulation of ABA gene function involves TF expression, which recognize and bind with cis-elements on the promoter region and upstream of its target genes [35]. In addition to the role of TFs, expression of ABA-responsive genes is also mediated by the secondary messenger, receptor, and protein kinase/phosphatase modules [33]. Studies have investigated the role of ABA cascades in MAPK-mediated signaling, including stomatal signaling and antioxidant defense in plants [36]. Consequently, interactions between various stress signaling pathways such as MAPK and ABA pathways are just beginning to be disclosed. The roles of ABA in MAPK-mediated signaling are reviewed above and in Figure 2.

## 3. Role of TFs in Drought Stress Signaling Pathways

TFs are the principal regulatory elements for many genes involved in environmental stress responses. TFs have vital roles in signaling pathways, from signal reception to the expression of genes related to drought stress in plants. Genes contain *cis*-acting components in their promoter regions, which serve as binding sites for TFs to regulate gene expression in signal transduction pathways. Signaling cascades in networks responsive to drought stress are activated via TFs that work together to induce drought tolerance [37]. Approximately, 1500 TFs are involved in the expression of stress related genes in *Arabidopsis* [38]. Several transcription factor families like MYB, WRKY, ERF, NAC, and bZIP have been characterized and shown to be useful tools for enhancing drought tolerance in plants. In recent studies, TFs involved in stress tolerance were identified in cotton and *Arabidopsis* (Table 1). Overexpression of *GhABF2* in cotton enhanced the activities of catalase (CAT) and superoxide dismutase (SOD), and improved yield in transgenic plants [39]. Another TF related to R2R3-type MYB, *GbMYB5*, responded positively to drought stress [40]. Ectopic expression of the *GhWRKY41* gene in tobacco plants led to increased activity of antioxidant enzymes, lower MDA content, increased stomatal closure, and upregulation of antioxidant-related genes [41]. In *Gossypium barbadense*, a R2R3-type *GbMYB5* TF gene enhanced drought tolerance in transgenic tobacco and cotton. These results suggest the involvement of GbMYB5 in adaptive drought stress responses [40]. GhWRKY59 is an important TF that ensures drought tolerance in cotton (Figure 2) [26]. In Upland Cotton, a NAM domain gene termed *GhNAC79*, improves drought tolerance, and also responds to JA and ethylene treatments. Additionally, its overexpression improved stress tolerance in *Arabidopsis* and cotton [42].

## 4. Cellular and Molecular Responses to Drought Stress in Plants

Drought stress affects plant growth, leaf and stem dry weights, canopy and root growth, plant height, and the number of nodes in plants. Similarly, some physiological properties, such as stomatal conductance, transpiration rate, photosynthetic rate, and water potential decrease under osmotic stress. Finally, osmotic stress limits the accumulation of dry matter by up to 50% under critical water deficiency [12,75]. These traits are potential candidates for drought tolerance in plants. Genetic improvement on the basis of physio-morphological traits is more important because, these traits have vital roles in maintaining a favorable water balance through stomatal closure, reduced transpiration, high water use efficiency, accumulation of proline, trihalose, and polyamines, leaf rolling, wax content, deep root system, and earliness [76].

**Figure 3:**
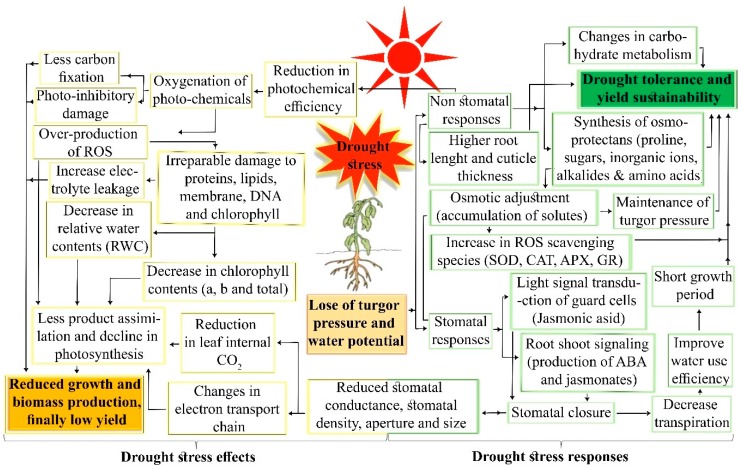
Overall pathways of drought stress effects and plant responses to drought stress.

After successful transduction of signals and sensing the drought stress, plants initiate drought recovery mechanisms through various physio-morphological and biochemical responses (Figure 3). Plants have developed various mechanisms to minimize or tolerate multiple stresses. Drought tolerance, drought recovery, drought escape, and drought avoidance are the four important categories of drought tolerance tools [10]. Tolerant plants subjected to stressful environments adopt an ‘escape scenario’ by utilizing energy for defense mechanisms, which eventually impacts growth and production. During drought avoidance, plants reduce transpiration and develop deep and vigorous root systems to increase water uptake to help maintain tissue water potential [77]. Tolerance to drought is the capacity of plants to endure severe dehydration through osmotic adjustment by osmo-protectants [77,78]. Drought recovery is the ability of plants to restart growth and overcome yield deficits following severe stress. Plants have established numerous morpho-physiological adaptations such as root growth, OA, photosynthetic rate, and stomatal regulation to overcome drought stress (Figure 3).

### 4.1. Morpho-Physiological Responses

Under drought stress, leaf rolling and wilting are important phenomena that help in regulating water loss in plants [79]. Xeromorphic traits in plants help to promote drought tolerance, and include thicker cuticles, thicker palisade tissue, epidermis, denser but smaller stomata, an improved vascular bundle-sheath, and thicker but smaller leaves [80]. Such xerophytic characters are also observed in a drought-tolerant variety of cotton, “YZ1,” which has smaller leaves than those of the drought-susceptible variety “Y668.” Gas exchange is a key mechanism used to maintain cellular functions to produce energy in plant tissues. Stomatal regulation plays a fundamental role in preventing water losses from stomata through transpiration, which often results in up to 90% water loss through stomatal opening [81]. When the transpiration rate increases, stomatal closure reduces water loss in cotton. In cotton, a negative correlation exists between stomatal conductance and drought tolerance, which is a potential marker of drought tolerance [3]. Stomatal regulation is a key mechanism for important cellular activities, which involves maintaining cellular water balance under drought stress environments.

The cellular effects of water deficiency include loss of osmotic balance and cellular turgidity. Thus, osmotic adjustment (OA) is a defense response that reduces the effects of drought in crops. Drought stress negatively affects the osmotic balance in plant cells [10]. Plants accumulate various inorganic and organic substances to maintain OA under drought stress. Several compounds, such as inorganic ions, sugars, sugar alcohols, amino acids, alkaloids, amines, and polyamines, which are osmolytes or osmoprotectants, are components of OA [82]. These solutes as well as the accumulation of high levels of inorganic ions help to protect cell membranes and proteins under drought stress [83]. Transgenic cotton for drought tolerance has increased OA capability, relative water content, photosynthesis, and lower ion leakage percentage. The expression of mustard annexin gene (*AnnBj1*) was shown to increase stress tolerance in cotton with higher sucrose and proline contents [84,85], whereas overexpression of the cotton *GhAnn1* annexin gene increased the action of SOD, proline, and soluble sugars for drought and salt tolerance [86].

Under stress conditions, CO_2_ intake is decreased due to stomatal closure, thus affecting photosynthesis and resulting in reduced growth and yield [87]. Gradual water deficit in plants fields affects growth and net photosynthesis under drought stress. Photosynthetically active young plants leaves are more heat and drought tolerant than older leaves. A 66% decline in photosynthesis was observed in older leaves, whereas no effect was observed in younger leaves at 37 °C. In two succeeding growth periods, a decline in lint production was observed due to lower net photosynthesis from water stress in cotton [11]. Potential functions of roots under drought stress have been reported in many studies, and several researchers have demonstrated interest in studying hydraulic conductance and plant allometry. Deeper roots and root density in soil are desirable traits for better adaptation to drought stress. Longer roots have been observed during the initial stages of drought stress in plants [88].

### 4.2. Biochemical and Cellular Responses

Plant biochemical compounds and their derivatives are key components in defense responses by plants against abiotic stresses. ABA is involved in several critical physiological processes throughout the life cycle of plants, including development, reproduction, and during stress responses. High drought and salt stress induce osmotic stress through the loss of turgor pressure. ABA promotes gene expression, which activates physiological changes that allow plans to adapt to stress condition [89]. When stress signals are received from the plasma membrane, ABA is synthesized in the plastids, except for the conversion of xanthoxin to ABA, which occurs in the cytoplasm. Roots are the main source of ABA, which is transported through the vascular channel to other parts of plant, mainly to the guard cells to enable stomatal closure [90,91]. Various ABA-dependent signaling transduction pathways have been established in plants. These pathways are responsible for stress tolerance via expression of stress-responsive genes (Figure 1, Figure 2 and Figure 3). With the overexpression of ABA-induced cotton genes *AREB1*, *AREB2*, and *GhCBF3*, drought tolerance increased as observed by the higher chlorophyll, proline, and relative water contents [6,92]. Drought tolerance has been positively correlated with activation of the ABA receptor gene, *GhPYL9-11A*, in transgenic plants [69]. Various ABA signaling and responsive genes are summarized in Table 1.

JA, which is derived from α-linolenic acid, is also involved in drought tolerance. Hence, α-linolenic acid is a source of JA and its other derivatives, termed jasmonates, also have important roles against many biotic and abiotic stresses. JA is involved in plant development and growth, viable pollen production, tendril coiling, fruit ripening, and root growth [93]. In a genomic analysis of cotton, various genes and molecular mechanism induced by JA, were involved in drought tolerance signaling pathways [94]. Participation of jasmonates in stomatal regulation is similar to the role of ABA [9]. Repressor proteins, such as jasmonate-zim domain (JAZ), act as a switch for the JA signaling pathway. Normally, in the absence of JA, jasmonate-zim (JAZ/JAI3) proteins bind with a number of TFs, such as myelocytomatosis (MYC2), and prevent their action. Conversely, during drought stress, in the presence of jasmonates, JAZ proteins are degraded and a TF (MYC2) activates stress-responsive genes [95]. Signal transduction of several assimilator processes is assisted by hormones, which work together to control various pathways involved in stress responses [9]. ABA and JA are core signaling components that process the response to the drought stress (Figure 1, Figure 2 and Figure 3). In the last few years, signaling pathways and JA biosynthesis have been reviewed comprehensively; however, the signaling pathways remain unclear.

### 4.3. Antioxidant Defense Against Cellular Reactive Oxygen Species (ROS)

Overproduction of ROS due to a reduction in atmospheric O_2_ is induced by various abiotic stresses in plants. ROS accumulation leads to cell death via progressive oxidative damage [96]. Four basic types of cellular ROS exist: hydrogen peroxide (H_2_O_2_), hydroxyl radical (HO^•^), singlet oxygen (^1^O_2_), and superoxide anion radical (O^2−^). HO• and ^1^O_2_ are highly reactive, and oxidize and damage several cellular components, such as DNA, RNA, proteins, and lipids. Eventually, uncontrolled oxidation causes cell death [10,96]. ROS is through photorespiration under drought stress conditions. Regulatory pathways and complex produced in the cell wall, plasma membrane, mitochondria, nucleus, and chloroplast [97]. Plants have developed the scavenging mechanisms to remove about 70% of H_2_O_2_, to maintain homeostasis of ROS redox reactions, and to avoid the overproduction of ROS [98]. Drought tolerance in plants can be influenced by modifications in antioxidant enzyme metabolism.

**Figure 4:**
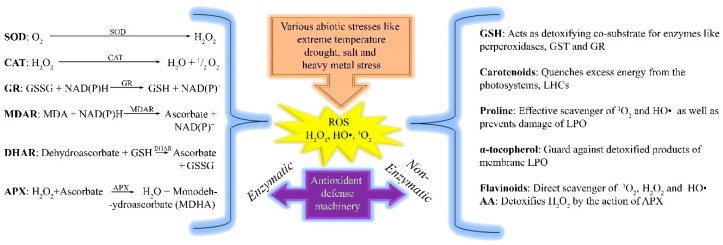
Anti-oxidant machinery scavenges cellular reactive oxygen species (ROS) through two pathways in plants. One is the enzymatic pathway and the other is a non-enzymatic pathway. Several enzymes convert ROS to non-harmful substances via enzymatic pathways in plant cells, while in the non-enzymatic pathway, other substances convert ROS to non-harmful substances.

Defense mechanisms in response to ROS have been reviewed to determine how plants have evolved antioxidant defense machinery for survival under water stress conditions. ROS scavenging mechanisms comprises of two pathways involving non-enzymatic antioxidants and enzymatic components. Non-enzymatic antioxidants include α-tocopherol, flavonoids, reduced glutathione (GSH), ascorbic acid (AA), carotenoids, and osmolyte-proline. These pathways work together to scavenge ROS. Enzymatic components include SOD, CAT, ascorbate peroxidase (APX), dehydroascorbate reductase (NADH), glutathione reductase (GR), guaiacol peroxidase (GPX), and monodehydroascorbate reductase (MDAR). MDAR, APX, GR, and NADH remove H_2_O_2_ through the ‘Halliwell–Asada’ pathway [99,100]. In the ascorbate–glutathione cycle, APOX converts H_2_O_2_ to H_2_O by oxidizing ascorbate to form MDHA [99,101]. Next, MDHA is reduced to ascorbate by MDHAR. Therefore, two MDHA molecules can be non-enzymatically transformed to dehydroascorbate and MDHA, and further condensed to ascorbate through the GR cycle and NADH [102] (Figure 4).

During drought stress, glutathione reductase activity is amplified to maintain an adequate ratio of reduced and oxidized glutathione (GSH). GSH is reduced by GSH reductase, an oxidation process that oxidizes GSH at the expense of NADPH in the GR cycle [103]. Oxidative signaling or damage depends on the balance between the production of antioxidant enzymes and ROS [75,104]. The tolerance of plant cultivars against drought stress depends on the activity of antioxidants. For example, ‘M-503’ tolerant cotton cultivar with active antioxidant enzymes (APX, SOD, POX, and CAT) reduce osmotic stress, followed by drought stress [105]. Another cotton cultivar ‘CCRI-60’ possesses ROS scavenging ability with increased growth compared to the drought-sensitive cultivar ‘CCRI-27’ [104]. In another study, the activities of antioxidant enzymes decreased with increasing oxidative stress due to the down regulation of *GbMYB5* in *Gossypium barbadense* [40]. ROS production triggers defense mechanisms associated with Ca^2+^ fluxes and ABA signaling (Figure 1 and Figure 2) [106]. Considering the ROS-activated defense machinery, both enzymatic and non-enzymatic scavenging events are likely to have vital roles in plant defense against drought stress.

## 5. Molecular Genetic Basis and QTLs for Drought Tolerance in Cotton

Various functional genes and genetic networks control complex agricultural traits in cotton. These genetic factors include minor and major QTLs with variable genetic interactions and regulation of several major genes. Such molecular modules display characteristics of a genetic functional unit, similar to breeding for specific molecular features that control stress-tolerance traits. Recently, a multiplex module was suggested for temperature-resilient crops [107]. Drought tolerance is complex; therefore, little improvement has been made through conventional breeding approaches. Molecular breeding techniques can supplement conventional breeding programs for crop improvement. Drought tolerance is a quantitative trait controlled by many loci, with each locus contributing a small effect. These loci are the genetic basis of several morpho-physiological reactions of plants, which represent the combined effects of hundreds of genes. In cotton, genetic information is limited, and phenotyping for drought tolerance is challenging. Only a few QTL linkage maps for drought have been identified in cotton. The first molecular map of cotton was generated using molecular markers (RFLP) in an interspecific cross between two species: upland and pima cotton using an F_2_ segregating population [108]. Subsequently, various types of molecular markers have been used in cotton to construct linkage maps through identification of QTLs responsible for yield, yield quality, and related traits under normal and stress conditions. These QTLs help us to understand the genetics of drought tolerance and other abiotic stresses. QTL mapping helps to locate these loci for marker-assisted breeding (MAS). Several QTLs have been mapped in cotton for morpho-physiological traits, including traits that contribute to yield, fiber traits, and earliness under drought-facing situations [109]. Different molecular markers are associated with the production of cotton under drought and normal circumstances [110].

Regarding the final fiber yield, several physiological, morphological, and biochemical traits related to abiotic stress have been studied. Previously reported that a leaf architecture type (Okra-leaf cotton) enhances drought tolerance by effecting early maturity and increasing photosynthesis by reducing leaf area in cotton [111]. Jiang used 261 restriction fragment length polymorphism (RFLPs) to map leaf morphology-related QTLs in a F2 population with 180 F2 plants from a hybrid cross between *Gossypium hirsutum* and *Gossypium barbadense*. Those authors mapped 40 QTLs, including an important locus related to drought stress on chromosome 6 that affects leaf trichrome density, and controls the rate of transpiration under water stress [112]. A phenotypic correlation was highlighted between physiology and yield-related traits in segregating populations of *Gossypium hirsutum* and *Gossypium barbadense* crosses [110]. Only lower osmotic adjustments were found to contribute to the high yield of seed cotton under water-limited conditions among the 33 identified physiological traits based on 253 RFLPs [110]. These QTLs have great scope for improving drought- and yield-related, polygenic traits through marker assisted selection (MAS).

A population comprising 188 F_2:3_, obtained from a hybrid crossed between *Gossypium hirsutum* and *Gossypium tomentosum*, was used to identify drought-related QTLs under field conditions [113]. Overall, 67 QTLs were identified under drought conditions, which were distributed on chromosomes 5, 8, 9, and 16. In this study mapping drought tolerance, all QTL results were inferred from populations of early segregants. Thus, development of a permanent mapping population is useful for various studies, and for the repeated use of the material for reliable detection of genetic variations. Consequently, an introgressed upland cotton population with permanent genetic makeup was developed using 1004 polymorphic DNA marker loci plus 523 single sequence repeats (SSRs) and 481 SNPs under both greenhouse and field conditions. Most common QTLs related to abiotic stress tolerance (drought and salt) were identified, and were distributed across 12 cotton chromosomes. The c5 chromosome contained a QTL cluster for plant height, which was observed under both field and greenhouse conditions for drought tolerance [2].

Different strategies are required for mapping QTLs related to drought tolerance, as a limited number of QTLs are linked to these stresses. Additional markers and candidate genes are required for abiotic stress tolerance in addition to these developments. Genome-wide SNP markers have been detected via genotyping-by-sequencing (GBS), a low cost alternative approach [114]. Another platform for SNP typing is the use and development of SNP chips for detection of high-level polymorphisms across large and diverse populations. Development of permanent genetic populations is needed for accurate QTL mapping in replicate experiments for the same population for drought tolerance. This becomes more important when exploring the genetic basis and variations that confer drought tolerance.

Thus, researchers have suggested, the use of large a recombinant inbred lines (RIL) population to study stress tolerance and genetic relationships [115]. A RIL population containing 146 lines was prepared to evaluate drought stress tolerance using PEG6000 under greenhouse conditions [116]. The same RIL population was used under field conditions to study drought tolerance. Both studies led to the identification of intervals between two STS (IH200-STS-IH590-STS) markers, which were linked to seed cotton, lint yield, uniformity and strength of fiber under field drought stress, and shoot weight (SW) under PEG stress. SSR marker intervals between the MUSS096 and MUSS009 regions were linked to lint percentage and fresh shoot-weight under field and PEG conditions, respectively. Another region, 1F470-1F480 marker interval was linked to fiber strength and plant height under field and PEG stress conditions, respectively [117]. Desirable drought-tolerant QTL alleles were present in a bi-parental inbred lines (BIL) population [118]. Moreover, there was a correlation between seed cotton and osmotic adjustment under stress. These findings reveal that favorable alleles for drought stress or other abiotic stresses can originate from either the tolerant *Gossypium barbadense* or the drought-sensitive *Gossypium hirsutum* [110]. Therefore, the possibility of species recombination to create new and favorable alleles expands the scope of genome-wide discoveries of complementary allele combinations.

Whole genome exposure has been achieved in cotton GWAS using cost-effective genome-wide DNA markers. Compared with a biparental mapping population, this method has high statistical power and resolution to detect major QTLs to explain the wide phenotypic variations observed. In GWAS, the population size is larger, such that in most studies, SSR markers are used in cotton genomes with low coverage. Very few studies have been published on drought tolerance, as phenotyping for drought tolerance is complicated in cotton. A GWAS performed using 106 SSR markers in 323 evaluated *Gossypium hirsutum* accessions led to the identification of 15 drought-tolerant and three salt-tolerant SSR markers with no overlap [119]. In GWAS, SNPs serve as candidates linking phenotype to genotype with genome-wide coverage. A GWAS was performed by using 26,301 polymorphic SNPs in 376 *Gossypium hirsutum* accessions to identify drought-tolerance QTLs. Several major and common QTLs were identified for biotic and abiotic stress tolerance, indicating that these QTLs may share common regions [120]. The main effects of QTLs for drought tolerance were detected based on shoot and root dry weight, which were further confirmed in another GWAS [121]. Those authors used 470,000 SNPs in 550 RILs and a MAGIC population of *Gossypium hirsutum*. These SNPs were derived from the reference genome sequence (TM-1). The authors identified 11 common QTLs between salt- and drought-stress tolerance among 16 and 27 QTLs for salt and drought tolerance, respectively, based on dry shoot weight and plant height. In another GWAS, 55,060 SNPs from a high-density cotton array (CottonSNP80K) were used in 319 *Gossypium hirsutum* accessions, and were phenotyped for nine drought stress-related traits. Twenty significantly-associated quantitative trait nucleotides (QTNs) and 205 drought-induced genes were found to be randomly distributed on 16 chromosomes [122]. 

Meta analyses can be performed to identify common QTLs by using shared markers from various QTLs from independent experiments. The first meta-analysis [123] identified common QTLs in various independent experiments in cotton. The authors compiled 1223 QTLs for yield, quality, disease, and drought resistance in a comprehensive analysis. However, they did not identify QTL hotspots or clusters for drought stress tolerance. Only a few drought-related QTLs were reported in cotton. A follow up meta-analysis was performed to detect hotspots and QTL clusters for drought tolerance among 661 stress-related QTLs, and 23 drought-tolerance QTLs were distributed on 15 different chromosomes. Two QTL hotspots associated with chlorophyll content were detected on chromosome c24 among the 28 different stress tolerance-related traits [124]. Newly-reported cotton QTLs can be easily grouped from all reported studies through meta-analyses. These results can be used for MAS to develop consistent drought-tolerant cotton varieties. Furthermore, theses QTL clusters and hotspots can be used to identify novel candidate genes responsible for drought tolerance. Various QTLs reported for drought tolerance traits are listed in Table 2. 

In MAS, QTLs can be used to explore the natural genetic variability of drought tolerance. Genetic mapping provides information on the location, numbers, degree, and pattern of gene action. Traditional methods of QTL assessment advanced genome mapping, and molecular MAS techniques have led to improvements in drought tolerance in plants. Cotton HY5-specific CAPS, dCAPS, PHYA, and PHYB molecular markers have been developed for MAS against drought stress [130]. MAS has been widely adopted over a phenotypic approach for better selection against drought tolerance, because of the unpredictable effects and the elusive nature of QTL for drought tolerance.

After exploring the complexity of drought tolerance, the next step is to interrogate selection tools to achieve drought tolerance in cotton. Cotton cultivars with drought tolerance have been developed conventionally by cotton breeders. Table 3 lists the drought-tolerant cotton genotypes that represent the principal genetic resources offering genetic variability for drought tolerance. BRS-286, CNPA-7MH, and CNPA-5M are drought-tolerant genotypes, and have been evaluated for antioxidant activity and growth traits [131]. Correlation between yield and antioxidant activity have also been observed [132]. Antioxidant activity, in addition to growth parameters, may be a valuable selection criterion. Giza 75, Suvin, and 10,229 drought-tolerant genotypes have been reported based on the drought stress index (DSI) along with high expression of the heat shock protein 1 gene (*GhSP1*) and flowering locus T-like protein 1 gene (*GhFTL1*) in tolerant genotypes, moderate expression in moderately-tolerant genotypes, and no expression in susceptible genotypes [133]. Gene expression is a precise and targeted selection criterion to improve drought tolerance, as it has favorable traits that are controlled by specific genes. Table 1 lists drought-specific candidate and major genes. Studies on their expression can help improve drought tolerance in cotton. Table 2 lists over 300 QTLs for physiological, morphological, and biochemical, drought-associated traits. These QTLs can be used to screen and develop drought-tolerant cotton verities through MAS. Genetic regions and major QTLs can also be employed to explore drought-tolerant candidate genes. In this new era of functional genomics, screening and assessment of tolerant cotton genotypes is more feasible and efficient.

## 6. Application of Genome-Modification Approaches to Achieve Drought Tolerance in Plants

Genetic engineering and biotechnology have made it possible to transfer desirable traits or genes between plants and species to obtain a desired phenotype. Transgenic approaches are useful for improving abiotic stress tolerance in plants following the discovery of numerous drought-tolerance genes. Many drought-tolerance genes have been overexpressed in cotton through various techniques (Table 4), and have enabled the transcription of such genes under drought stress conditions. Overexpression of the *Arabidopsis AtEDT1/HDG11* (enhanced drought tolerance 1/homeodomain glabrous 11) gene resulted in enhanced proline content, more dynamic ROS scavenging activity, an extensive rooting system, and improved drought tolerance in cotton [145]. Another study highlighted the performance of transgenic cotton carrying the *IPT* gene, as it has better drought tolerance, in the event of stress prior to flowering stage [146]. Ectopic endogenous and exogenous overexpression of two abiotic stress-responsive TF orthologs (bZIP AREB/ABF) from *Gossypium hirsutum*, *GhABF2D* and *Arabidopsis AtABF3* were studied. Drought resilience substantially increased in transgenic *Gossypium hirsutum*, primarily through improved stomatal regulation [147]. Although transgenic approaches hold promise, some challenges, such as the long incubation times and low transformation efficiency. exist in cotton.

The CRISPR/Cas9 system is a combination of two parts: a clustered regularly interspaced short palindromic repeat and an associated protein 9, found in bacteria (*S. pyogenes*). It has been effectively used in various model plant species for fast and targeted genome editing [157]. Discovery of the CRISPR/Cas9 system was a breakthrough, and it serves as a multipurpose tool for gene alteration in plants. The first application of genome editing by CRISPR/Cas9 in plants has been successful in the model plant, *Arabidopsis* [158]. However, there have been few reports on the successful application of CRISPR/Cas9 in cotton. Recently, multiple targeted genome editing in allotetraploid cotton by targeting *GhCLA1* (chloroplast development gene) and GhARG (arginase discosoma red fluorescent protein 2) genes showed its adaptability for genome editing in cotton [159]. Replacing a native promoter locus of ARGOS8 by the native *ARGOS8* gene in maize results in the production of high yield under drought stress [160]. In the promoter region of genes, cis-sequences are essential regulatory elements of genes and various abiotic stress responses. The role of these cis-sequences in stress regulation has also been documented [161]. Various cis-regulatory sequences are negative regulators of drought stress tolerance, such as W-box (TTGACC), which provide a binding site for TF *GhWRKY17* in cotton [60]. These represent candidate regulatory sites for targeted mutations with the CRISPR/Cas9 system. Recently, targeting these cis-sequence sites through the CRISPR/Cas9 system was proposed to create new QTLs for analysis of sustainable phenotypic and genetic variations, which can contribute to improve abiotic stress tolerance in plants such as cotton [162]. Several candidate target genes are summarized in Table 1, and include the WRKY and DREB TF gene family, which can be modified to regulate drought tolerance in cotton. However, technical challenges and low transformation efficiency limit its extensive use in cotton.

## 7. New Functional Genomic Tools to Identify Novel Genes for Stress Tolerance in Plants

Sequencing of tetraploid cotton genomes [163], sub-genomes and other *Gossypium* sp., including *Gossypium ramondii* [164], *Gossypium barbadense* [165], and *Gossypium arboreum* [166], and recently, genome-wide re-sequencing of 352 cotton accessions [167], 243 diploid accessions [168], and 419 accessions [169] present a comprehensive genome-wide assessment to identify genes, SNPs, and genomic regions, which are useful to select for abiotic and drought stress tolerance in cotton. Sequencing and re-sequencing of cotton genomes provide a foundation to identify genes and genome structures that describe the biology of cotton. Advancements in cotton functional genomics will aid to exploration of biologically-active regions and genes from the entire genome. Currently, we are able to study SNP array platforms, fine and high-density genetic maps, transcript abundance, and epigenetic modifications for drought stress tolerance in cotton. Subsequently sequencing of sub-genome species, and the construction of ultra-precise and dense genetic linkage maps will provide a platform for gene mapping, isolation, and high-throughput marker development for stress tolerance [14]. Furthermore, here we report 300 QTLs identified exclusively for drought tolerance from interspecific and intraspecific populations of *Gossypium hirsutum* and *Gossypium barbadense* in cotton (Table 2). However, the identified QTLs contain several genes and large genomic regions, which may be helpful for MAS. Nevertheless, fine maps of genomic regions carrying various markers to enhance the selection efficiency, will aid the isolation of specific genes present at a particular locus.

Whole-genome (2.5 Gb) SNPs have been developed in allotetraploid cotton with advances in NGS techniques and in silico methods. In cotton, development of the SNP63K array containing 17,954 and 45,104 putative interspecific and intraspecific SNP marker assays, respectively, is valuable [170]. This provides a high-throughput genotyping platform, a basis and standard tool for genetic analyses of stress-related, agronomically, and economically important traits in cotton. A significant proportion of the whole genome is covered by copy-number-variations (CNVs) instead of SNPs. However, CNVs can be useful for identifying phenotypic variations for complex traits [14] including, abiotic stress tolerance, which are normally not covered by SNPs. CNVs in plant genomes can modify gene dosage, regulation, structure, and they affect genes related to several abiotic stress tolerance and agronomic traits [171]. CNV alterations in 989 genes have been identified in the cotton genome, and are related to translational regulation, plant cell wall organization, and plant type [167].

Transcriptome profiling is an important tool that to extract information through sequence data to gain knowledge on various gene functions and pathways. Recently, the whole-genome transcriptome was reported which provides information about expressed sequence tag (EST) assemblies of TM-1 inbred line (*Gossypium hirsutum*), and serves as a reference genome for all RNA-sequence-based SNP studies [170]. Transcriptome libraries of *Gossypium barbadense* for stress-related traits such as drought, salt, heat, cold, and phosphorus, were also standardized as a reference to identify novel stress-related genes [172]. Transcriptome analysis for drought and abiotic stresses in cotton has been conducted by RNA-Seq. analysis of large-scale gene expression through tetraploid and diploid genome sequences and NGS technologies [173]. In a comparative transcriptome analysis, the expression pattern of differentially expressed genes (DEGs) revealed the transcription of stress-related genes induced by environmental stress in somatic embryos [174]. However, some challenges exist for RNA-Seq, such as the need to process and store large data sets and handle library construction.

Among the genetic factors, the epigenetic based modifications also regulate the various gene functions in plants. Among the various epigenetic signaling approaches, DNA methylation is the most common, as it plays a significant role in the evolution of morpho-physiological diversity in plants. Seasonal variation in DNA methylation based modifications have been observed for fiber development and other plant tissues in cotton [175,176,177,178]. DNA methylation plays a dynamic role in development of the fiber and ovule, CHH methylation, and dependency of RdDM (RNA-directed DNA methylation) has been observed for the activation of various genes in ovules. Chromomethylase 2 (CMT2)-dependent DNA methylation silences some genes in growing fiber [179]. Recently, epigenetic modifications were utilized to modify 519 cotton genes in domesticated cultivars and wild species. Few genes were also related to domestication and agronomic characters [180]. DNA methylation s is involved in regulating the immune system of *Arabidopsis thaliana*, particularly against biotic stress [181]. These findings provide an understanding of epigenetic regulation, modifications of the polyploid evolution and development of different domesticated traits in cotton. MicroRNAs are short noncoding RNAs (miRNAs) involved in post-transcriptional gene expression and regulation by translational repression or mRNA degradation. In cotton, the evolution of miRNA-coding genes was investigated along with their role in ovule and fiber development. They play vital roles in the gene expression and regulation of various biological processes including metabolism, cell profiling, development, and stress responses [182]. These studies can clarify how cotton miRNAs interact with stress-related genes under variable environments.

## 8. Functional Genomics for Stress Tolerance

Genome-wide comparative analysis of expression profiling in plants revealed that different molecular signatures (transcripts, transcriptional factors, and genes), physiological, and biochemical processes work in association to induce drought tolerance. Various TFs ensure stress signaling responses by regulating the expression of several upstream and downstream genes related to the mechanism of stress tolerance in plants. Thus, TFs are excellent candidate genes to enhance drought tolerance in plants [183]. Functional genomic approaches are useful to assign specific functions to genes sorted from stress-related candidate genomic regions, QTLs, and DEGs, which are involved in stress-tolerance mechanisms. In cotton, only a few genes have been verified through true-to-type assigned functions and experimental validation. Their expression and adaptability for drought tolerance in cotton is unclear. 

**Figure 5:**
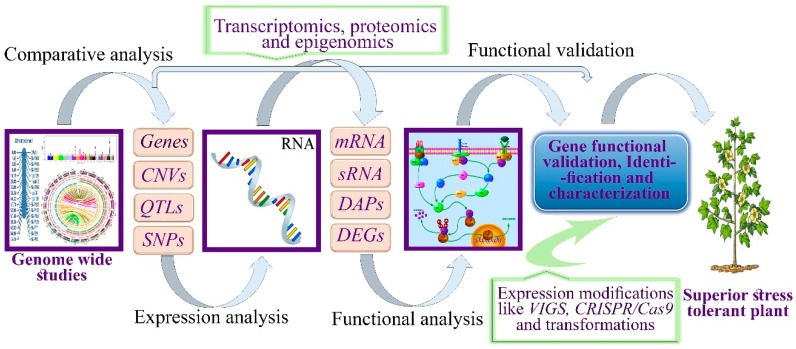
Schematic flow of research processes from genome-wide studies to functional validation, characterization, and identification of drought stress-responsive genes in plants. QTL, quantitative trait loci, CNV, copy number variation, SNP, single nucleotide polymorphism, mRNA, messenger RNA, SRNA, small RNA, DAP, differential abundant proteins, and DEG, differential expressed genes.

Transcriptome sequences and genome sequencing do not provide insight into the specific role of genes. Previously, gene functions have been elucidated through their annotation and comparison of sequences with other genomes of model plants, without performing wet experiments. Comparative analysis does not reveal the functional basis of genes and the biologically active states of a genome under specific stress conditions without experimental verification. Advancements in genomic studies in cotton will reveal the biologically active and functional sites of DNA. Cotton has been proposed as an alternative model crop for other polyploids and high-density genetically diverse species [184]. Transcript abundance, fine maps, epigenetic modifications, and SNP array platforms were studied across multiple species and tissues. Whole genome sequencing [185], resequencing, and the high efficacy of NGS technologies offers a new foundation and considerable data analysis platform. Here, we proposed a third-generation sequencing approach coupled with functional genomic tools. Using this approach, sequencing data including genome-wide resequencing, transcriptomics sequencing, proteomics sequencing, and epigenomic sequencing data can be combined to identify the major candidate genes and biologically active states of DNA involved in several defense mechanisms in plants (Figure 5).

In an era of emerging science and technology, application of high-throughput sequencing provides new ways to exploit the diverse genetic basis. This method demonstrated the physio-molecular foundation of stress-related influences through sequencing and resequencing of various cotton species and their phenotyping. Polyploidy and evolution-based duplication in the cotton genome explains the limited success with genomic tools, limited understanding of agronomic traits such as productivity, and quality deterioration of cotton fiber due to climate change and global warming (heat and drought). Tetraploid cotton fibers have better emergent properties than diploid cotton under the same environmental conditions [185]. This divergence in coordinate expression due to the proximal functioning of distinct genes such as transposable elements under different regions and environmental conditions [186]. Recently sequenced genomes of members of the Malvaceae provide a basis for thos diverged and coordinated expression. Recently sequenced genomes of the Malvaceae family include those of *Hibiscus syriacus* [187], *Theobroma cacao* [188], *Gossypium hirsutum* [163], *Gossypium barbadense* [165], *Gossypium ramondii* [164], and *Gossypium arboreum* [166]. It is useful for comparative studies on the evolution and migration of different cotton species globally. Knowledge of cotton genomics is limited compared to that of other model plants.

Various novel tools and approaches have been used in plants to minimize the adverse effects of drought (Figure 5). Regardless of previously reported improvements, opportunities exist for the improvement of drought tolerance in plants. Good knowledge of plant biology, physiology, field performance, root architecture, stomatal conductance, osmotic adjustments, and photosynthesis along with other metabolic processes in cotton plants are needed. These are important attributes for enhancing drought tolerance in plants. Future studies should be complemented with advanced biotechnological and molecular approaches to understand plant responses to drought stress. Genomic sequencing, transcriptomics, proteomics, and bioinformatic analyses can help to address the complexity of drought tolerance.

## 9. Future Perspectives and Conclusions

In this review, we explored the mechanisms of cellular stress signaling in plants and the genetic basis of drought tolerance in cotton. We provided a broad picture of recent advancements and understandings of various stress signaling pathways in plants. Additionally, drought-induced, hormone-dependent MAPK signaling and interactions between ABA, ROS, and MAPK signaling pathways are comprehensively discussed. Various mechanisms of cellular stress tolerance in plants seem to be interconnected and their degree of association is influenced by environmental factors. The molecular genetic basis and foundations of these mechanisms have not been fully explored due to the complexity and phenotyping difficulties of drought tolerance. Additionally, transposable genetic elements and epigenetic modifications, particularly DNA methylation, are also sources of variation of stress tolerance in plants. Regardless of the previous improvements, huge potential still exists for improving tolerance in plants. The latest genetic information is required to understand plant biology, cell physiology, and plant-environment interactions. Similarly, exploration of genetic factors related to the root architecture, stomatal conductance, osmotic adjustments, and photosynthesis in plants would be of value. Sequencing and re-sequencing of the cotton genome offers a comprehensive genome-wide assessment to identify genes, SNPs, and QTLs, which serve as a signature for drought tolerance. Given such advances, we can now study SNP array platforms, fine- and high- density genetic maps, transcript abundance, and epigenetic modifications for stress tolerance in cotton, as performed for other model plants. Subsequently, the sequencing of sub-genome species, and ultra-precise and dense genetic linkage maps provides a platform for gene mapping, isolation, cloning, and high-throughput marker development. Here, we propose a third-generation sequencing approach in addition to functional genomic tools, which can be applied to combine all sequencing data including genome-wide resequencing and post-transcriptional sequencing (proteomics, transcriptomic, and epigenomic) to sort the entire set of genes and biologically active states of DNA involved in drought tolerance mechanisms. Future studies should be complemented with integrated and multidisciplinary approaches to understand plant and environment interactions. Advances in plant stress tolerance rely on multidisciplinary functional genomic approaches, including genomics, epigenomics, proteomics, transcriptomics, and bioinformatic analysis. The true-to-type gene function should be verified through wet experiments rather than simple annotation and sequence comparisons. The molecular design of plant breeding is an integrated, goal-oriented approach, consisting of several modules combined with various disciplines, including synthetic biology, system biology, genomic biology, and computational biology. Computational biology involves molecular modules for specific targeted traits, which can be used to transfer a specific trait into a specific genome of target plant variety and used as a basic framework for synthetic biology for more manipulations. Dissection of molecular modules with a strong genetic basis and natural variations disclose the relevant modules that control the focused traits. These methods can be combined with genome modification tools, such as transgenic technologies and the CRISPR-Cas9 system, as well as additional diverse genetic resources containing selection elements based on categorized molecular modules, QTLs, and key rolling genes. The proposed technological strategies can also be employed for drought tolerance in cotton and other crops. Thus, the above discussed approaches and techniques serve as an efficient platform and network to understand the complexity of stress tolerance in plants.

## Figures and Tables

**Table 1:** Key genes involved in abiotic stress signaling in rolling cotton.

Gene	Type	Phenotypic Effect/Function	Reference
*GhHUB2*	Histone H2B monoubiquitinatin E3 ligase encoding gene	Drought tolerance through increased soluble sugar, proline, and leaf relative water contents	[43]
*GrMAPKKK* and *GhMAPKKK*	MAPK gene family	Drought and salt responsive	[20]
*GhMAP3K1*, *GhMKK4*, and *GhMPK6*	MAPK signaling gene	Regulates the drought stress response by interacting with *GhWRKY59–GhDREB2*	[26]
*GhMKK3*	MAPK signaling gene	Enhanced drought tolerance	[25]
*GhMAP3K40*	MAPK signaling gene	Salt and drought stress tolerance at the germination stage	[44]
*GhMPK4*	MAPK signaling gene	Increased sensitivity to ABA, salt, and drought	[45]
*GhMPK17*	MAPK signaling gene	Osmotic and salt stress tolerance	[46]
*GbMPK3*	MAPK signaling gene	Enhanced oxidative and drought stress tolerance	[47]
*GhMPK6a*	MAPK signaling gene	Drought and salinity	[48]
*GhMKK1*	MAPK signaling gene	Drought and salinity	[49]
*GhMKK5*	MAPK signaling gene	Drought and salinity	[50]
*GhMPK2*	MAPK signaling gene	Drought and salinity	[51]
*GbRLK*	Receptor-like kinase	Drought and salinity	[52]
*GaHDG11* *(HD-ZIP)*	Transcription factor	Drought and heat stress	[53]
*GhNAC79*	Transcription factor	Improves resistance to drought stress	[42]
*GhERF38*	Transcription factor	Drought, abscisic acid, and salinity	[54]
*GhERF2*, *GhERF3*, *GhERF6*	Transcription factor	Drought, salt, ethylene, and abscisic acid	[55]
*GhWRKY59*	Transcription factor	Activates MAPK signaling gene under drought	[26]
*GhWRKY25*	Transcription factor	Drought and salinity	[56]
*GhABF2 (bZIP)*	Transcription factor	Enhances the activities of CAT and SOD, regulates gene expression related to ABA	[39]
*GhNAC2*	Transcription factor	Longer roots, and enhanced salt and drought tolerance	[57]
*GhCBF3*, *GhAREB1*, and *GhAREB2*	ABA-induced gene	Small stomatal aperture, enhanced drought- and high salinity-tolerance via the ABA signaling pathway	[58]
*GhNAC7-GhNAC13*	Transcription factor	Cold, abscisic acid, drought, and salinity	[59]
*GbMYB5*	Transcription factor	Reduced water loss trough stomatal conductance, and increased proline content and antioxidant enzymes	[27]
*GhWRKY41*	Transcription factor	Lower malondialdehyde content, higher antioxidant activity, and induced stomatal conductance	[41]
*GhWRKY17*	Transcription factor	Increases sensitivity to ABA and drought stress	[60]
*GhNAC8-GhNAC17*	Transcription factor	Drought, salinity, cold, and ABA	[61]
*GhNAC1-GhNAC6*	Transcription factor	Drought, cold, salinity, and ABA	[62]
*GhDREB*	Transcription factor	Drought, cold, and salinity	[63]
*GhDREB1*	Transcription factor	Drought, cold, and salinity	[64]
*GhDBP2*	Transcription factor	Drought, cold, and ABA	[65]
*GhERF1*	Transcription factor	ABA production and drought stress signaling regulation	[66]
*GhERF4*	Transcription factor	ABA production and drought stress signaling regulation	[67]
*GhDREB1L*	Transcription factor	Drought, cold, and salinity	[68]
*GhPYL9–11A*	ABA receptor gene	ABA receptor that mediates the response to drought stress	[69]
*GhSnRK2*	Involved in ABA signaling	Drought, salinity, cold, and ABA	[70]
*GhCDPK35*, *GhCDPK28*, *GhCDPK16*, *GhCDPK14*, *GhCDPK11* and *GhCDPK3*	Ca^2+^-activated gene	Drought and salinity stress responsive	[7]
*GhCIPK6*	Ca^2+^-activated gene	Increased drought, salinity, and ABA stress tolerance	[30]
*GhD12G207*	CDK gene family	Increased concentration of antioxidant enzymes (POD, SOD, and CAT), cell membrane stability, and chlorophyll content under drought and salt stress	[71]
*GaMYB62L*	Transcription factor	Increased chlorophyll and proline contents, higher germination rate under drought salt stress	[72]
*GhTPS11*	Functional gene	Drought, heat, salinity, ABA, and gibberellin acid	[73]
*GhAVP1*	Functional gene	Drought and salinity tolerance	[74]

**Table 2:** Quantitative trait loci (QTLs) of drought tolerance in cotton.

QTL	Traits	Size and Type of Population	Number and Types of Markers Used	Reference
49	Lint yield, seed cotton yield, fiber length, fiber elongation, boll weight, leaf area, fresh shoot weight, and plant height	97 F5:9 RILs (TM-1 × NM24016)	RGA-AFLP, SSR and GBS-SNP (1004)	[125]
59	Canopy temperature, normalized difference vegetation index, canopy height, and leaf area index	95 RIL (TM-1 × NM24016)	SSR (429)	[126]
67	Plant height, chlorophyll content, leaf number, leaf area, leaf dry and fresh weights, number of fruiting branches, number of bolls, and boll weight	188 F_2:3_ (CRI-12 × AD3-00)	SSR (1295)	[113]
6	Plant height, and fresh shoot and root weight.	142 BILs (Pima S-7 × Sure-Grow747)	AFLP, RGA and RGA-AFLP (34)	[127]
14	Chlorophyll content, leaf temperature, fresh shoot and root weight, evapotranspiration, and plant height	140 RILs (Dan-dara × Giza-70)	SSCP (165)	[116]
3	Excised leaf water loss and relative water content	100 F2 (B-557 × FH1000)	SSR and EST-SSR (524)	[109]
6	Relative water content, excised leaf water loss, cell membrane stability, stomatal frequency, and stomatal size	100 F_2_ (FH-207 × FH901)	EST-SSR (2365)	[128]
7	Chlorophyll content, osmotic potential, carbon isotope ratio, and seed cotton yield	28 NILs (GH ‘Sivon’ × GB cv.F-177)	RFLP (279)	[81]
79	Chlorophyll a and b, carbon isotope ratio, osmotic potential, canopy temperature, dry matter, harvest index, boll weight and boll number, and seed cotton yield	208 F3 (GH ‘Sivon’ × GB cv.F-177)	RFLP (253)	[110]
3	Osmotic potential, osmotic adjustment, and plant height	136 F2 and F2:4 (FH-901 × RH-510)	SSR (6500)	[129]

**Table 3:** Reported drought-tolerant cotton genotypes and the genetic basis for drought tolerance.

Genotypes	Origin	Traits/Method	Reference
06K485, 06K486, SPAN 837, FQMA (05)5bcp, Chureza, and RASAM 17	DARS, Malawi	Fresh and dry root weight, lateral roots number, tap root length, root volume, fresh and dry shoot weight, stem diameter, shoot length, and number of leaves per plant	[134]
GhAM-46, GhAM-9, EC560413, and GhAM-78	India	SPAD chlorophyll contents, excised leaf water loss, root volume, root and shoot length, root and shoot weight, and final yielding	[135]
LRA-5166, BS-37, CCH-12-3, BS39, GBHV-177, GBHV-182, and ARBH-1352	India	Root and shoot length, percent seed germination, and seedling vigor (shoot vigor index, seedling vigor index, and root vigor index)	[136]
BRS 286, CNPA 7MH, and CNPA 5M	Brazil	Antioxidant enzymes activities (APX, CAT, and SOD)	[131]
H1353/10 × G.Cot.16 and G.Cot.16 × H-1353/10	India	Yield index, yield stability index, yield reduction ratio, mean productivity, geometric mean productivity, stress susceptibility index, tolerance index, and stress tolerance index	[137]
Giza75	Egypt	Drought stress index (DSI) and expression of drought-related genes (Gossypium heat shock protein 1 [*GhSP1*] and flowering locus T-like protein 1 [*FTL1*])	[133]
Suvin	India		
10229	Australia		
Giza80, Giza90, Giza80 × Tamco C.E., Giza90 × (Giza9 × Giza Australian) and Giza90 × TamcoC	Egypt	Enzymatic (ascorbate peroxidase, catalase, peroxidase, and superoxide dismutase) and non-enzymatic (phenolic content, lipid peroxidation, and proline) antioxidant activities	[132]
Acala-1517-99, DAK-66/3, and GC-555	USA	Seed germination, seedling growth, yield, yield components, and genotypes characterized with low drought susceptibility index, and high geometric mean productivity	[138]
Nieves	Australia		
MS-30/1 and Nazilli M-503	Turkey		
Eva and Zeta 2,	Greece		
NIAB-999	Pakistan		
Delta Diamond	Spain		
Sindh-1 and Shahbaz-95	Pakistan	Lint yield per plant, boll weight, bolls per plant, sympodial branches per plant, and plant height	[139]
FH-942 and FH-113,	Pakistan	Excised leaf water loss, shoot and root lengths, number of lateral roots, fresh root and shoot weights (g), dry shoot and root weight (g), and total plant fresh weight (g)	[140]
MARVI, CRIS-9, CRIS-, CRIS-337, CRIS-126, CRIS-355, and 377CRIS-134	Pakistan	Chlorophyll content, RWC, transpiration rate, excised leaf water loss, yield components, and yield	[141]
FH-113, MNH-789, and PB-899	Pakistan	Chlorophyll, carotenoids, and polyphenols	[142]
149F, BOU 1724-3, B-557, and DPL-26	Pakistan	Drought tolerance indices, relative shoot and root length	[143]
Acala-1517–99 and CRS-M-9044–0165	USA	Seedling traits	[144]

**Table 4:** Transgenes overexpressed in cotton for drought tolerance.

Gene	Effects on Cotton Drought Tolerance	Effect on Yield	Stress Type	Donor Specie	References
*AtABF3*	Improved stomatal regulation, less transpiration, and photosynthetic productivity	Yield increased	Drought	*Arabidopsis thaliana*	[147]
*IPT*	More number of bolls and larger root systems	Yield increased	Drought and heat	*Agrobacterium tumefaciens*	[146]
*OsSIZ1*	Higher net photosynthesis, better growth, and cotton fiber yield	Yield increased	Drought and heat	Rice	[148]
*ScALDH21*	Soluble sugar and proline content increased, higher peroxidase activity, reduced loss of net photosynthesis, reduced lipid peroxidation, greater plant height, and larger bolls	Yield increased	Drought	*Syntrichia caninervis*	[149]
*AtEDT1/HDG11*	Soluble sugar and proline content increased, well-developed roots, low stomatal density, increased ROS scavenging enzymes	43% more seeds	Drought and salt	*Arabidopsis thaliana*	[145]
*AtNHX1 and AVP1*	Plant height, boll number, and fiber yield	24–35% more fiber	Drought and Salt	*Arabidopsis thaliana*	[150]
*SNAC1*	Enhanced proline content and root development, decreased transpiration rate	31% more bolls	Drought and salt	Rice	[151]
*SNAC1*	Reduced transpiration rate and more vigorous root system		Salt and drought	Rice	[151]
*AtABI5* & *AtRAV1/2*	ROS scavenging, osmotic adjustment, improved photo-assimilation, root and shoot sink strengths, enhanced expression of *GhRAV* and genes for antioxidant and osmolyte biosynthesis	Yield affected	Drought	*Arabidopsis thaliana*	[152]
*AtLOS5*	Enhanced ABA levels to improve drought tolerance with 13% more fresh biomass	13% more fresh biomass	Drought and heat	*Arabidopsis thaliana*	[153]
*AVP1*	Enhanced sequestration of ions and sugars into the vacuole, reduced water potential, and enhanced root biomass	Increased 20%	Drought and salt	*Arabidopsis thaliana*	[154]
*TsVP*	Improved root and shoot growth, higher rate of photosynthesis and relative water content, less cell membrane damaged	27–53% higher in Lumianyan2142–61% in Lumianyan19	Drought	*Thellungiella halophila*	[155]
*betA*	Increased photosynthesis, higher relative water content, better osmotic adjustment, less ion leakage, and lipid membrane peroxidation	3–12% higher	Drought	*Escherichia coli*	[85]
*GF14λ*	Higher photosynthesis rate, enhanced senescence, and chlorophyll content	Enhanced	Drought tolerance	*Arabidopsis thaliana*	[156]
